# Clinical and economic burden of achondroplasia in the United States: results from a retrospective, observational study

**DOI:** 10.1186/s13023-024-03268-w

**Published:** 2025-02-27

**Authors:** Nadia Merchant, Jose Alvir, Paulette Negron Ericksen, Jane Loftus, Jose Francisco Cara, Alison Slade, Michael P. Wajnrajch, Christine L. Baker

**Affiliations:** 1 Division of Pediatric Endocrinology, Department of Pediatrics, University of Texas Southwestern Medical Center, Dallas, TX USA; 2 Pfizer Inc., New York, NY USA; 3 Pfizer Walton Oaks, Tadworth, UK; 4 Pfizer AG, Zurich, Switzerland; 5 New York University Grossman School of Medicine, New York, NY USA

**Keywords:** Achondroplasia, Comorbidities, Pediatrics, Adults, Healthcare resource utilization, Costs, Economic burden

## Abstract

**Background:**

Achondroplasia, a disease characterized by disproportionate short stature and increased morbidity, affects daily function and quality of life over the lifetime of the individual. However, data are limited on its economic impact, especially related to healthcare resource utilization (HCRU) and associated costs. This study aimed to characterize the clinical and economic impact of achondroplasia in the US relative to matched non-achondroplasia controls stratified by pediatric and adult populations.

**Methods:**

This retrospective study used data from the IQVIA PharMetrics Plus national claims database from January 2008 to December 2021. Individuals diagnosed with achondroplasia (index event) between July 2008 and December 2020 were matched on age and sex (1:2 ratio) to non-achondroplasia controls. General comorbidities were evaluated in the pediatric and adult populations. All-cause HCRU and direct medical costs were determined for the 12-month post-index period; out-of-pocket (OOP) costs were also determined. Study variables were analyzed using descriptive statistics.

**Results:**

A total of 530 individuals with achondroplasia (47.7% pediatric and 52.3% adults) were matched with 1,060 controls. Individuals in the achondroplasia cohort had higher overall comorbidity burdens than controls. HCRU was higher in the achondroplasia cohort relative to controls, with outpatient visits the most frequently used resource. Inpatient visits were the primary driver of mean (SD) total costs, which were 14-fold higher than controls ($28,386 [$259,858] vs $2,031 [$5,418]) in pediatric individuals, and 4-fold higher in adults $21,579 [$58,817] vs $4,951 [$13,020]); prescriptions accounted for 4.7% and 7.4% of total costs in the pediatric and adult achondroplasia cohorts, respectively. The OOP costs were approximately 3-fold higher in both pediatric and adult individuals with achondroplasia relative to controls.

**Conclusions:**

Individuals with achondroplasia are characterized by a higher comorbidity burden and substantially higher HCRU and related costs relative to matched controls. The results also suggest that despite high HCRU and costs, individuals with achondroplasia likely are not seen by providers early enough nor are they necessarily seen by appropriate specialists, indicating a need for improved care and disease management.

**Supplementary Information:**

The online version contains supplementary material available at 10.1186/s13023-024-03268-w.

## Background

Skeletal dysplasia refers to a group of rare genetic disorders that result in abnormal development of bones, joints, and cartilage. Achondroplasia is the most common form of skeletal dysplasia caused by gain-of-function variant in either G380R or G375C in the gene for the fibroblast growth factor receptor 3 (*FGFR3*), resulting in reduced and disproportionate growth due to defective long bone ossification centers in the cranium and axial skeleton [1]. Estimates suggest that the birth prevalence of achondroplasia is 4.7 per 100,000 births worldwide and 4.0 per 100,000 births in North America [2].

Individuals with achondroplasia are characterized by short stature, macrocephaly, frontal bossing and midface retrusion, rhizomelic shortening of limbs, and kyphosis. Serious complications affect different organ systems and can include spinal stenosis, sleep-disordered breathing, otitis media, hearing loss and speech delay, and chronic pain [3, 4]. Medical management has traditionally focused on complications resulting from abnormal skull, spine, and extremity ossification with interventions that have included surgical decompression and arthroscopic surgeries [3]. Development of pharmacologic disease-modifying therapies for potential use in the treatment of achondroplasia has relied on mechanistic approaches that target the FGFR3 pathway directly or the C-natriuretic peptide pathway, which regulates longitudinal bone growth. Such approaches include small molecule tyrosine kinase inhibitors (infigratinib), monoclonal antibodies (vofatamab), soluble decoy receptors (recifercept) against FGFR3, and long-acting analogues (vosorotide, BMN 111) or pro-drugs (TransCon CNP) of C-natriuretic peptide [5]. Currently, only vosorotide is approved based on a pivotal phase 3 clinical trial that showed a benefit in growth and good tolerability after 52 weeks of treatment versus placebo with continued improvement at two-year follow-up [6, 7]. However, evaluation of longer term tolerability and extended effects on clinical and patient-reported outcomes in individuals treated with vosorotide is still needed [8], and availability of additional pharmacologic therapies that target the underlying cause of achondroplasia would likely benefit this population.

The clinical, functional, and psychosocial challenges that develop and vary with age in individuals with achondroplasia warrant appropriate management across the lifespan and suggest the need for coordination of multidisciplinary and/or interdisciplinary care. Published management recommendations and updates have mainly targeted the pediatric population [9–11], and only recently have broader consensus guidelines been developed that reflect a multidisciplinary approach to follow-up and monitoring from infancy through adolescence and adulthood [12, 13].

In addition to substantial morbidity [4, 14], the life span of individuals with achondroplasia has been reported to be 10 years shorter than the general population [15]. However, over the past years, survival has been improving, especially in the pediatric achondroplasia population [16], likely resulting from greater disease recognition and improved clinical management. This increase in survival emphasizes the need for a more holistic approach over the lifetime of the individual.

While achondroplasia has a substantial impact on physical function, including impairment of activities of daily living, and reduced health-related quality of life [4, 17–20], a literature review highlighted that data on its economic impact is limited [21], and only a single study evaluated costs associated with achondroplasia [22]. That study, conducted in the United States using nationally representative databases, reported on inpatient encounters and outpatient surgeries, but utilization and costs of other healthcare resource categories were not considered (e.g., outpatient visits, prescriptions, emergency room, etc.), nor did it include a control cohort, although national averages were presented to provide context. Among the findings was that the overall average achondroplasia hospital admission was two days longer than the national average, and almost $8,000 more expensive, with estimated annual costs from the societal perspective of $40 million.

Given the dearth of data on healthcare resource utilization (HCRU) and related costs associated with achondroplasia, the objective of this study was to provide a more comprehensive perspective on these variables by characterizing the clinical and economic impact of achondroplasia in the US relative to matched controls (i.e., non-achondroplasia cohort) stratified by pediatric and adult populations. In particular, we wanted to determine the prevalence of comorbidities and evaluate all-cause HCRU (medication use, healthcare visits, procedures) and their associated costs as well as out-of-pocket (OOP) costs for these individuals.

## Methods

### Study design and data source

This retrospective, observational, matched cohort study used claims data from the IQVIA PharMetrics Plus claims database for the study period January 2008 to December 2021. PharMetrics Plus is a national claims database of commercial health plans that includes de-identified records of more than 215 million individuals in all 50 states since 2006. It consists of adjudicated inpatient and outpatient medical claims and outpatient pharmacy claims with month-by-month information on enrolled patients. The database is compliant with data security requirements of the Health Insurance Portability and Accountability Act (HIPAA) of 1996, and since the study did not entail collection, use, or transmittal of identifiable data, it was exempt from the requirement for institutional review board approval.

### Population

Identification of the achondroplasia cohort was based on ≥ 1 International Classification of Diseases (ICD) diagnosis codes for achondroplasia between July 2008 and December 2020. These codes included ICD-10-CM Q77.4 as well as ICD-9-CM 756.4 prior to October 1, 2015, with confirmation based on the subsequent ICD-10-CM code after October 1, 2015. The first diagnosis code was defined as the index event.

Achondroplasia individuals were matched to controls, i.e., no achondroplasia diagnostic code, in a 1:2 ratio based on age and sex. Both cohorts were required to have continuous enrollment 6 months pre- and 12 months post-index. A cancer diagnosis during the pre-/post-index period was reason for exclusion in both cohorts. Given that non-achondroplasia controls will not have a corresponding index date, after matching for age and sex, a “hypothetical index date” was considered with an enrollment window for the potential controls confirmed to be at least as long as that of the achondroplasia individual. If there were sufficient data, i.e., all enrollment criteria and available data requirements are met, the controls were considered to be a match and two were randomly selected; any non-selected controls were re-entered into the pool for potential matching with a subsequent individual with achondroplasia.

### Variables and outcomes

Demographic variables included sex, age, geographic region, and insurance type; age was used to identify pediatric and adult subpopulations and further stratify the pediatric and adult subpopulations into age groups

The comorbidity burden during the 6-month pre-index period was evaluated in the pediatric population using the Pediatric Comorbidity Index (PCI) [23] and in the adult population using the Quan-Charlson Comorbidity Index (CCI) [24], with the individual comorbidities contained within these indices identified based on ICD codes. The 10 most common comorbidities during this time period were also identified in the pediatric and adult populations according to Clinical Classifications Software Refined (CCSR).

All-cause HCRU was determined for the 12-month post-index period by frequency of use (i.e., proportion of individuals using each resource category) and units of use. Categories evaluated were prescriptions, outpatient visits, home health care visits, emergency room (ER), inpatient, and surgeries (defined as any surgical intervention regardless of whether inpatient or outpatient). Outpatient visits were also stratified by specialty and included pediatrics, radiology, otolaryngology, orthopedic, general/family practice, anesthesiology, other, and unknown. Additionally, Current Procedural Terminology codes were used to identify common surgical procedures.

Direct medical costs over the 12-month post-index period were estimated as the mean standard allowed charges and are expressed in 2021 dollars. Costs were for all-cause HCRU and included inpatient and non-ER outpatient services as well as ER and prescriptions, with total costs that were the sum of these categories. The OOP costs for these individuals were also estimated, and were defined as the difference between the amount paid by the plan and the allowed charges.

### Statistical analysis

Study variables were analyzed using descriptive statistics including means for continuous variables and number and frequency for categorical variables; 95% confidence intervals (CI) were determined for HCRU and cost data. For the analyses, the cohorts were stratified by age groups, with the pediatric population defined as those < 18 years old and stratified as 0 to < 2 years, 2 to < 6 years, 6 to < 11 years, 11 to < 18 years, and adults defined as ≥ 18 years old and stratified as 18 to < 41 years, 41 to < 65 years, and ≥ 65 years. All analyses were conducted using SAS version 9.4 (SAS Institute, Cary, NC).

## Results

### Population characteristics

Among 205,760,936 individuals in the database for the study period, 2,435 had an index diagnosis of achondroplasia of whom 530 met all other criteria for inclusion and were matched with 1,060 non-achondroplasia controls (Additional file 1: Table S1). The cohorts were 44.2% male and consisted of 47.7% pediatric and 52.3% adult patients, predominantly with commercial insurance (58.1%-60.3%) (Table 1).

**Table 1:** Demographic characteristics

Variable	Achondroplasia cohort (*n* = 530)	Control cohort (*n* = 1,060)
Sex, n (%)		
Male	234 (44.2)	468 (44.2)
Female	296 (55.9)	592 (55.9)
Age, years		
Mean (SD)	24.7 (19.0)	24.7 (19.0)
Median (Q1, Q3)	19 (9, 39)	19 (9, 39)
Age group, years, n (%)		
< 2	24 (4.5)	48 (4.5)
2 to < 6	53 (10.0)	106 (10.0)
6 to < 11	77 (14.5)	154 (14.5)
11 to < 18	99 (18.7)	198 (18.7)
18 to < 41	152 (28.7)	304 (28.7)
41 to < 65	116 (21.9)	232 (21.9)
≥ 65	9 (1.7)	18 (1.7)
Geographic region, n (%)		
East	103 (19.4)	190 (17.9)
Midwest	162 (30.6)	274 (25.9)
South	163 (30.8)	379 (35.8)
West	101 (19.1)	197 (18.6)
Unknown/missing	1 (0.2)	20 (1.9)
Insurance type, n (%)		
Commercial	308 (58.1)	639 (60.3)
Medicaid	50 (9.4)	90 (8.5)
Medicare	17 (3.2)	4 (0.4)
Self-insured	150 (28.3)	321 (30.3)
Missing/unknown	5 (0.9)	6 (0.6)

*Q1, Q3* first and third quartiles, *SD* Standard deviation

### Comorbidities, all-cause HCRU, and associated costs

#### Pediatric population

Among the 253 pediatric individuals with achondroplasia, the comorbidity burden was higher relative to the 506 pediatric controls as indicated by mean (SD) PCI scores of 0.95 (1.61) and 0.36 (1.02), respectively. In the achondroplasia cohort, 40.3% had any of the PCI comorbidities vs 20.2% of controls. The PCI comorbidities that were more prevalent in the achondroplasia cohort relative to controls were congenital malformations (18.2% vs 2.2%), developmental delays (6.7% vs 1.4%), joint disorder (3.2% vs 1.0%), sleep disorders (3.2% vs 0.4%), and anemia (1.2% vs 0%).

Among the 10 most frequent CCSR comorbid conditions in the achondroplasia cohort, five overlapped with the 10 most frequent among controls (Additional file 1: Fig. S1) Among the 5 overlapping conditions, otitis media and respiratory signs and symptoms had a higher prevalence in the achondroplasia cohort, 6.7% vs 4.7%, and 5.5% vs 3.4%, respectively.

**Fig. 1:**
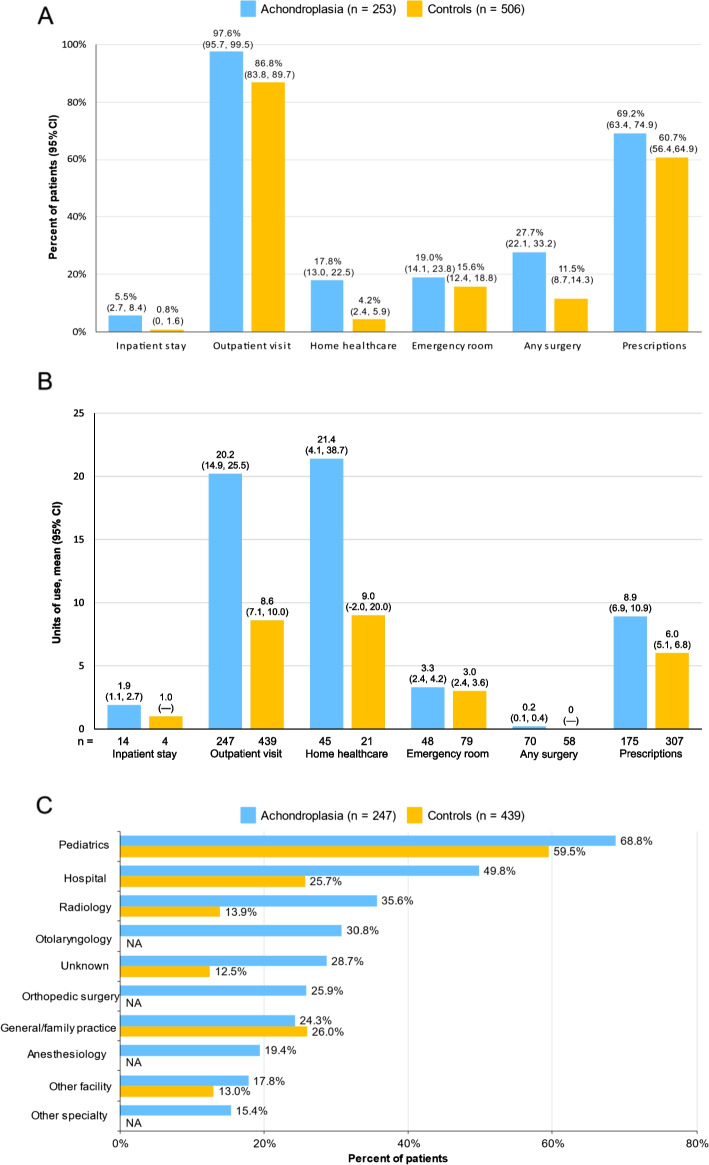
Healthcare resource utilization in the 12-month follow-up period among pediatric individuals with achondroplasia and matched controls. **A** Resource categories. **B** Units of resource use. **C** The 10 most frequently seen outpatient visit specialties in the achondroplasia cohort among pediatric individuals with outpatient visits. Values for controls in panel C reflect specialties in the top 10 for this group, with NA indicating “not available” but imputed as < 8% based on the 10^th^ most frequently seen specialty

Use of all categories of healthcare resources was higher in the achondroplasia cohort (Fig. 1A). The most frequently used resource category was outpatient visits, 97.6% (95% CI: 95.7, 99.5) in the achondroplasia cohort and 86.2% (95% CI: 83.8, 89.7) in controls, followed by prescriptions, 69.2% (95% CI: 63.4, 74.9) and 60.7% (95% CI: 56.4, 64.9) in the two cohorts, respectively. While the proportion with inpatient visits was approximately 7-fold higher in the achondroplasia cohort relative to controls (5.5% [95% CI: 2.7, 8.4) vs 0.8% [95% CI: 0, 1.6]), those with achondroplasia also had substantially higher use of home healthcare (17.8% [95% CI: 13.0, 22.5] vs 4.2% [95% CI: 2.4, 5.9]) and surgery (27.7% [95% CI: 22.1, 33.2] vs 11.5% [95% CI: 8.7, 14.3]). Units of use were higher in the achondroplasia cohort for all resource categories except ER, which was similar between the cohorts (Fig. 1B); home healthcare and outpatient visits were the categories with the highest utilization per individual in both groups. Mean length of inpatient stay was 8.9 days (SD 23.0; 95% CI -4.4, 22.2) in the achondroplasia cohort and 2.0 days (SD 0.8; 95% CI: 0.7, 3.3) in controls. Of the 10 most commonly seen outpatient specialties in the achondroplasia cohort, most of these specialist visits were also among the 10 most commonly seen specialties by controls, although controls had a lower rate of utilization except for general/family practice, which was the only category with an incrementally higher rate (Fig. 1C). Orthopedic surgery and anesthesiology were among the top 10 specialties in the achondroplasia cohort, 26.6% and, 19.7%, respectively, but not in controls (Fig. 1C).

The most frequent surgical procedures in the achondroplasia cohort were related to ear, nose, and throat, and included tympanostomy (8.3%), removal of impacted cerumen (4.0%), and tonsillectomy/adenoidectomy (3.2%). Destruction of benign lesions was the most frequent surgical procedure in the control cohort (1.8%), with all other procedures, most of which appeared to be related to injuries, occurring in <1% of individuals (data not shown).

When HCRU was stratified by age groups (Additional file 1: Fig. S2), rates of use were substantially higher in the achondroplasia cohort relative to controls across age groups for inpatient visits, home healthcare, and any surgery. Outpatient visits were similar to or incrementally higher in the achondroplasia cohort relative to controls, and ER use was higher in the achondroplasia cohort among those 0 to < 2 years and 6 to < 11 years but was similar to controls in the other age groups. Use of prescription medications was incrementally higher in the achondroplasia cohort for all pediatric age groups except 0 to < 2 years old. Among the individuals with achondroplasia, inpatient stays were higher in the younger age groups, and while the percentage with outpatient visits was similar across age groups, home healthcare, ER, and surgeries were highest among those aged 0 to < 2 years and tended to decrease with increasing age (Additional file 1: Fig. S2). The proportion of individuals with prescription medication use was generally similar across age categories. The number of units of use of each resource category varied by age, with higher units of use generally observed in the achondroplasia cohort; the exceptions were for home healthcare and ER, which were both higher in the controls for individuals 6 to < 11 years old, and prescriptions, which were similar in the two cohorts for individuals 6 to < 11 years old (Additional file 1: Fig. S3). The only clear age-related trends appeared to be higher use of inpatient stays, outpatient visits, and any surgery in the younger age groups of the achondroplasia cohort.

Mean (SD) total costs were approximately 14-fold higher in the achondroplasia cohort than controls, $28,386 (SD $259,858; 95% CI: $-3,789, $60,561) vs $2,031 (SD $5,418; 95% CI: $1,559, $2,504) (Table 2), with inpatient costs the primary cost driver in the achondroplasia cohort, accounting for 71.6% of total costs; non-ER outpatient visits was the primary cost driver in the control cohort (69.0% of total costs). Prescription costs comprised < 5% of total costs in the achondroplasia cohort, but accounted for 17.5% of total costs in controls.

**Table 2:** All-cause healthcare resource utilization costs in the 12-month follow-up period among pediatric individuals with achondroplasia and matched controls

**Healthcare resource**	**Mean cost ± SD (95% CI)**	
	**Achondroplasia** **(*n* = 253)**	**Non-achondroplasia controls** **(*n* = 506)**
Inpatient	$20,336 ± 256,685 (-11,446, 52,118)	$73 ± 1,108 (-24, 170)
ER	$247 ± 837 (144, 351)	$202 ± 942 (119, 284)
Non-ER outpatient visit	$6,867 ± 19,312 (4,475, 9,258)	$1,402 ± 4,283 (1,028, 1,776)
Prescription	$960 ± 4,483 (405, 1,515)	$356 ± 2,301 (155, 557)
Total	$28,386 ± 259,858 (-3,789, 60,561)	$2,031 ± 5,418 (1,559, 2,504)

*CI* Confidence interval, *ER* Emergency room, *SD* Standard deviation

When stratified by age, costs in the achondroplasia cohort were consistently and substantially higher than controls across pediatric age groups and resource categories (Additional file 1: Table S2). The highest total costs in the achondroplasia cohort were among those 0 to < 2 years old ($172,602 [SD $831,390; 95% CI: $-171,972, $530,159), and while these costs were driven by inpatient costs that accounted for 94.5% of the total, the large SD indicates that a small subset had high inpatient utilization. Costs in other age groups in the achondroplasia cohort, as well as in controls, were primarily driven by non-ER outpatient costs.

The OOP costs were ~3.5-fold higher among the achondroplasia cohort relative to controls ($1,522 [SD $3,879; 95% CI $1,042, $2,003] vs $439 [SD $974; 95% CI $353, $524), and were generally similar across age groups.

#### Adult population

In the adult population, the mean CCI suggested an overall greater comorbidity burden in the achondroplasia cohort than the control cohort, 0.26 (0.86) and 0.15 (0.49), respectively. Five of the 10 most prevalent CCSR comorbidities in the achondroplasia cohort overlapped with the top 10 comorbidities of controls, and all had a higher prevalence than in controls (Additional file 1: Fig. S4). Among these comorbidities, the achondroplasia cohort had more pain-related conditions than controls including musculoskeletal other than low back pain (14.8% vs 9.4%), spondylopathy/spondyloarthropathy (11.2% vs 5.8%), low back pain (9.4% vs 4.5%), and headache/migraines (7.2% vs 3.3%).

**Fig. 2:**
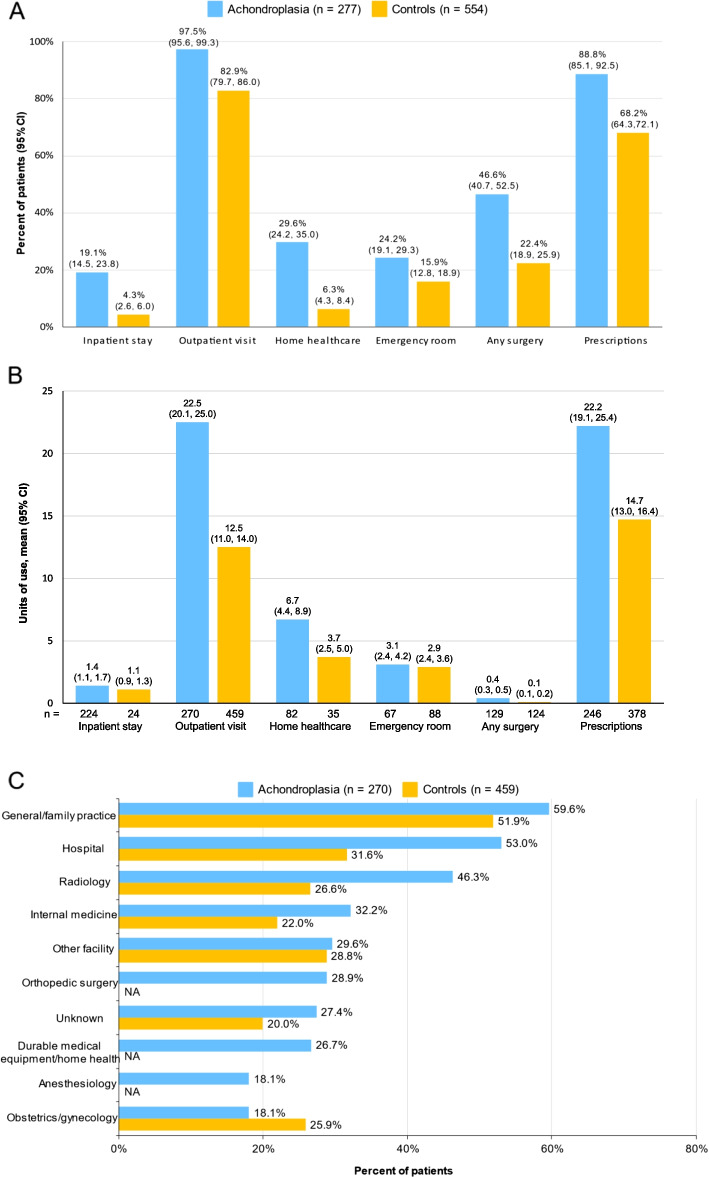
Healthcare resource utilization in the 12-month follow-up period among adults with achondroplasia and matched controls. **A** Resource categories. **B** Units of resource use. **C** The 10 most frequently seen outpatient visit specialties in the adult achondroplasia cohort among individuals with outpatient visits. NA, not available but < 8% based on frequency of the 10 most frequently seen specialties in the control group

Across all healthcare resource categories, the proportion of individuals who used these resources was consistently higher in the achondroplasia cohort relative to controls (Fig. 2A). While the most frequently used resource category was outpatient visits, almost 1 out of 5 individuals with achondroplasia had an inpatient stay for a rate that was more than 4-fold higher than controls (19.1% [95% CI: 14.5, 23.8] vs 4.3% [95% CI: 2.6, 6.0]. Units of use among individuals who used each resource was substantially higher for outpatient visits and incrementally higher for other resource categories (Fig. 2B). Most of the 10 most frequently seen outpatient specialties in the achondroplasia cohort were also observed among controls who had lower rates of utilization except for obstetrics/gynecology, which was higher among the controls (25.9% vs 18.1%) (Fig. 2C). Mean length of inpatient stay was 5.0 days (SD 4.9; 95% CI 3.6, 6.3) and 2.3 days (SD 2.5, 95% CI: 1.2, 3.3) in the achondroplasia and control cohorts, respectively, and individuals with achondroplasia were also more likely than controls to have an inpatient readmission within 30 days (3.6% [95% CI: 1.4, 5.8] vs 0%), 90 days (4.0% [95% CI: 1.7, 6.3] vs 0%), and 365 days (4.7% [95% CI: 2.2, 7.2] vs 0.2% [95% CI: 0, 0.5]) after the first admission (data not shown).

The three most common surgical procedures among adults in the achondroplasia cohort were “arterial catheterization or cannulation for sampling, monitoring, or transfusion” (7.2%) followed by “arthrocentesis, aspiration, and/or injection, major joint or bursa, without ultrasound” (4.3%) and “laminectomy, facetectomy, foraminotomy” (3.2%). In the control cohort, the three most common procedures were “arthrocentesis, aspiration, and/or injection, major joint or bursa, without ultrasound” (2.3%), “esophagogastroduodenoscopy, flexible, transoral; with biopsy” (2.3%), and “colonoscopy, flexible, with biopsy” (1.1%).

Higher rates of HCRU across resource categories were also observed in the achondroplasia cohort relative to controls when stratified by age (Additional file 1: Fig. S5). While all individuals with achondroplasia who were ≥65 years old had ER, surgeries, and prescription medications, these individuals had a low rate of inpatient stays, albeit this age group was characterized by a small number of individuals. Units of outpatient visits was substantially higher in the achondroplasia cohort relative to controls across age categories, but other resource categories were generally similar between the cohorts or incrementally higher, and were characterized by wide variability in units of use (Additional file 1: Fig. S6).

Mean total costs were approximately 4-fold higher in the achondroplasia cohort than controls, $21,579 (SD $58,817; 95% CI: $14,622, $28,536) vs $4,951 (SD $13,020; 95% CI: $3,864, $6,037), with these costs driven by inpatient costs and non-ER outpatient costs in the two cohorts, respectively (Table 3). While prescription costs were similar between the achondroplasia and control cohorts, they comprised 7.4% and 25.3% of total costs, respectively. Similar results were observed when stratified by age (Additional file 1: Table S3); total costs in the achondroplasia cohort were 3.3-4.5-fold higher relative to controls, with inpatient costs and non-ER outpatient costs the primary cost drivers in the achondroplasia and control cohorts, respectively, across all age groups. The highest total costs in the achondroplasia cohort were for individuals 41-<65 years old, driven by the high inpatient costs relative to the other age groups.

**Table 3:** All-cause healthcare resource utilization costs in the 12-month follow-up period among adults with achondroplasia and matched controls

**Healthcare resource**	**Mean cost ± SD (95% CI)**	
	**Achondroplasia** **(*n* = 277)**	**Non-achondroplasia controls** **(*n* = 554)**
Inpatient	$13,699 ± 54,459 (7,258, 20,141)	$944 ± 6,076 (437, 1,451)
ER	$392 ± 1,375 (229, 555)	$216 ± 761 (152, 279)
Non-ER outpatient visit	$5,955 ± 8,692 (4,927, 6,983)	$2,558 ± 6,528 (2,013, 3,103)
Prescription	$1,608 ± 5,149 (999, 2,217)	$1,251 ± 5,625 (782, 1,721)
Total	$21,579 ± 58,817 (14,622, 28,536)	$4,951 ± 13,020 (3,864, 6,037)

*CI* Confidence interval, *ER* Emergency room, *SD* Standard deviation

The OOP costs were almost 3-fold higher among the achondroplasia cohort relative to controls ($2,805 [SD $9,770; 95% CI: $1,650, $3,961] vs $970 [SD $2,385; 95% CI: $772, $1,170]). The mean OOP costs were generally similar among the age groups in the achondroplasia cohort and were higher than controls (data not shown).

## Discussion

This retrospective claims analysis shows that individuals with achondroplasia are characterized by more comorbidities, increased HCRU, and a substantially greater economic burden than matched controls. These results indicate the magnitude of the burden relative to controls and also highlight the cost differences between pediatric and adult populations with achondroplasia; pediatric individuals with achondroplasia not only had higher absolute costs than adults, but these costs were proportionally greater relative to controls than for the adult populations.

While the resource category with the highest utilization was outpatient visits, hospitalizations represent the resource with the highest unit cost and was the primary cost driver in both the pediatric and adult achondroplasia cohorts. The pediatric population had a small proportion of inpatient stays (5.5%) but a subpopulation with long length of stay that likely resulted in the large variability of inpatient costs. In contrast, 20% of the adult achondroplasia cohort had at least one inpatient stay, and readmission was more likely than among the matched controls. However, in both the pediatric and adult achondroplasia cohorts, prescription costs comprised only a small percentage of total costs, likely reflecting the lack of pharmaceutical interventions that are available for the treatment of achondroplasia, as the time period of the study was prior to approval of vosoritide.

Consistent with previous studies [20, 22], pediatric and adult individuals with achondroplasia saw a range of outpatient specialties and received surgical procedures that reflected age-specific differences in the needs of these individuals. The differences in type and frequency of common surgical procedures between the achondroplasia and control cohorts are further indicative of the substantial differences in resource utilization and the needs of individuals with achondroplasia. Similar to what has been reported by Broder et al. [22], ear, nose, and throat procedures were common among the pediatric individuals with achondroplasia and musculoskeletal procedures were more common in the adults with achondroplasia. Such resource use emphasizes that care is dependent on a multidisciplinary or interdisciplinary team and is consistent with recommendations that a multidisciplinary/interdisciplinary approach is integral to providing support across the individual’s life span [12, 13]. Although it has been previously reported that there is a bimodal distribution in medical complications and utilization of resources, which are most frequently seen in the youngest and oldest age groups [20], such distributions were not consistently observed in the current study. Nevertheless, use of some of these resources appeared to be low, especially in the pediatric population, suggesting that these individuals likely are not seen by providers early enough. These results potentially indicate a need for improved age-appropriate patient care and disease management.

Of additional relevance to these individuals is that OOP costs were significantly higher than those of the matched controls, suggesting that individuals with achondroplasia bear a substantial economic burden related to direct medical costs. In this regard, the economic burden borne by individuals and their families may be underestimated, as indirect costs and costs related to informal care were not estimated; further evaluation of the family burden of achondroplasia is warranted.

### Limitations

The use of ICD codes for identification of achondroplasia may be considered a study limitation, as the ICD-10-CM diagnosis code for achondroplasia is also used for hypochondroplasia and congenital osteosclerosis and thus has the potential for misclassification in some individuals. Another limitation is the inability of claims to capture clinical information such as disease severity, disease duration, or progression that likely contribute to resource use and costs. Additionally, only direct medical costs and OOP costs were evaluated. Consequently, the overall economic burden is likely to be underestimated, as costs related to use of aids, home modification, informal care, formal care/paid caregiving, and transportation were not considered, nor were indirect costs related to lost work productivity (individual and caregiver). The burden may additionally be underestimated because the time period of the study was during the COVID-19 pandemic, likely resulting in decreased HCRU.

## Conclusions

Patients with achondroplasia are characterized by a higher comorbidity burden and substantially higher HCRU and related costs relative to matched controls, with a difference between cohorts that is especially notable in the pediatric population. Despite the high rate of HCRU across resource categories, individuals with achondroplasia likely are not being seen early enough by healthcare providers, nor are these individuals necessarily receiving age-appropriate screening and management. While the results suggest a need for improved care and management across the life of the individual with achondroplasia, more attention should be paid to the pediatric population, which could not only improve the lives of these individuals but may also potentially reduce costs and improve morbidity over their lifetime.

## Supplementary Information

Additional file 1: Fig. S1. Prevalence of the 10 most common Clinical Classifications Software Refined comorbidities in the pediatric achondroplasia cohort (A) and pediatric non-achondroplasia controls. (B) Comorbidities in bold font indicate overlap between cohorts. Fig. S2. Healthcare resource utilization in the 12-month follow-up period among pediatric individuals with achondroplasia and matched controls stratified by age. A) Inpatient stays. B) Outpatient visits. C) Home healthcare. D) Emergency room. E) Any surgery. F) Prescriptions. Fig. S3. Units of healthcare resource use in the 12-month follow-up period among pediatric individuals with achondroplasia and matched controls stratified by age. A) Inpatient stays. B) Outpatient visits. C) Home healthcare. D) Emergency room. E) Any surgery. F) Prescriptions. Fig. S4. Prevalence of the 10 most common Clinical Classifications Software Refined comorbidities in the adult achondroplasia cohort compared with the prevalence extracted from the 20 most frequent CCSR comorbidities in adult non-achondroplasia controls. Comorbidities in bold font indicate overlap with the top 10 among controls. Fig. S5. Healthcare resource utilization in the 12-month follow-up period among adults with achondroplasia and matched controls stratified by age. A) Inpatient stays. B) Outpatient visits. C) Home healthcare. D) Emergency room. E) Any surgery. F) Prescriptions. Fig. S6. Units of healthcare resource use in the 12-month follow-up period among adults with achondroplasia and matched controls stratified by age. A) Inpatient stays. B) Outpatient visits. C) Home healthcare. D) Emergency room. E) Any surgery. F) Prescriptions. Table S1. Population attrition. Table S2. All-cause healthcare resource utilization costs in the 12-month follow-up period among pediatric individuals with achondroplasia and matched controls stratified by age groups. Table S3. All-cause healthcare resource utilization costs in the 12-month follow-up period among adults with achondroplasia and matched controls stratified by age groups.

## Data Availability

Data that support the findings of this study are available from Pfizer, but restrictions apply to the availability of these data, which were used under license for the current study and are not publicly available. Data are, however, available from the authors upon reasonable request and with permission of Pfizer. The authors can confirm that relevant data are included in the article.
