# Supplementary material for: Clinical and economic burden of achondroplasia in the United States: results from a retrospective, observational study
Source: Orphanet J Rare Dis. 2025 Feb 27;20:90. doi: 10.1186/s13023-024-03268-w (PMC11869590; doi:10.1186/s13023-024-03268-w)
Supplement: Supplementary file 1 — Additional file 1: Fig. S1. Prevalence of the 10 most common Clinical Classifications Software Refined comorbidities in the pediatric achondroplasia cohort (A) and pediatric non-achondroplasia controls. (B) Comorbidities in bold font indicate overlap between cohorts. Fig. S2. Healthcare resource utilization in the 12-month follow-up period among pediatric individuals with achondroplasia and matched controls stratified by age. A) Inpatient stays. B) Outpatient visits. C) Home healthcare. D) Emergency room. E) Any surgery. F) Prescriptions. Fig. S3. Units of healthcare resource use in the 12-month follow-up period among pediatric individuals with achondroplasia and matched controls stratified by age. A) Inpatient stays. B) Outpatient visits. C) Home healthcare. D) Emergency room. E) Any surgery. F) Prescriptions. Fig. S4. Prevalence of the 10 most common Clinical Classifications Software Refined comorbidities in the adult achondroplasia cohort compared with the prevalence extracted from the 20 most frequent CCSR comorbidities in adult non-achondroplasia controls. Comorbidities in bold font indicate overlap with the top 10 among controls. Fig. S5. Healthcare resource utilization in the 12-month follow-up period among adults with achondroplasia and matched controls stratified by age. A) Inpatient stays. B) Outpatient visits. C) Home healthcare. D) Emergency room. E) Any surgery. F) Prescriptions. Fig. S6. Units of healthcare resource use in the 12-month follow-up period among adults with achondroplasia and matched controls stratified by age. A) Inpatient stays. B) Outpatient visits. C) Home healthcare. D) Emergency room. E) Any surgery. F) Prescriptions. Table S1. Population attrition. Table S2. All-cause healthcare resource utilization costs in the 12-month follow-up period among pediatric individuals with achondroplasia and matched controls stratified by age groups. Table S3. All-cause healthcare resource utilization costs in the 12-month follow-up period among adults [file 13023_2024_3268_MOESM1_ESM.docx]

**Additional File 1**

**Fig S1.** Prevalence of the 10 most common Clinical Classifications Software Refined comorbidities in the pediatric achondroplasia cohort (A) and pediatric non-achondroplasia controls (B). Comorbidities in bold font indicate overlap between cohorts.


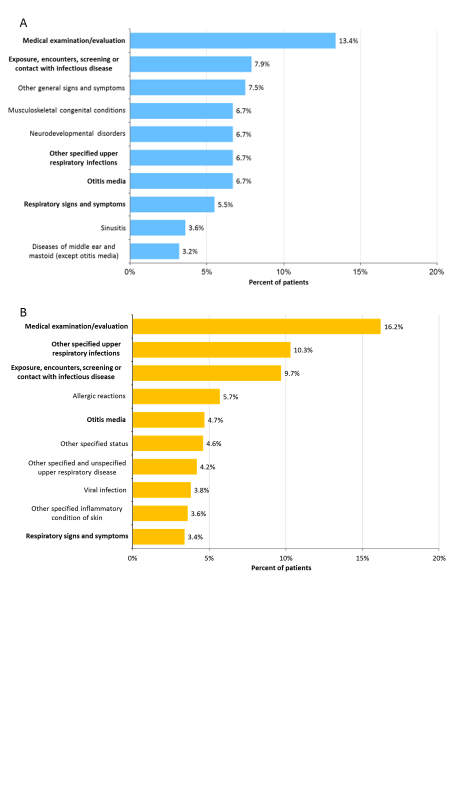


**Fig S2.** Healthcare resource utilization in the 12-month follow-up period among pediatric individuals with achondroplasia and matched controls stratified by age. A) Inpatient stays. B) Outpatient visits. C) Home healthcare. D) Emergency room. E) Any surgery. F) Prescriptions.

**
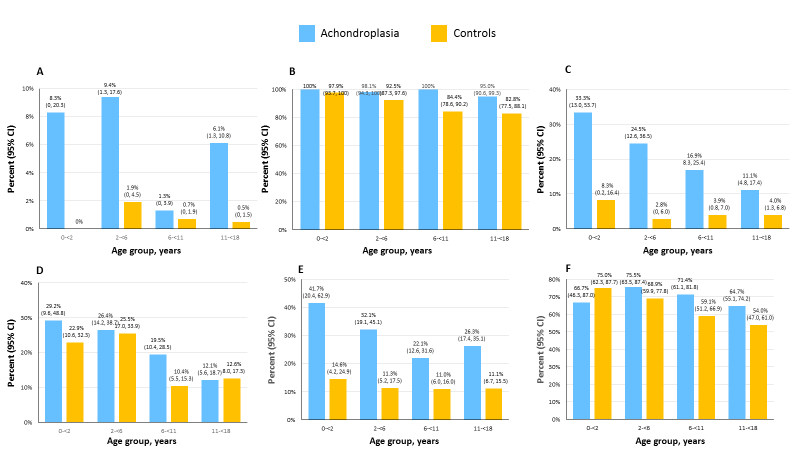
**

**Fig S3.** Units of healthcare resource use in the 12-month follow-up period among pediatric individuals with achondroplasia and matched controls stratified by age. A) Inpatient stays. B) Outpatient visits. C) Home healthcare. D) Emergency room. E) Any surgery. F) Prescriptions.

**
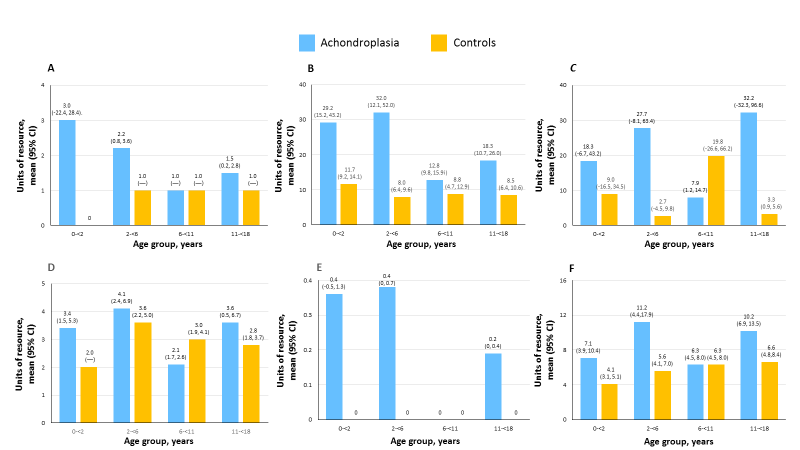
**

**Fig S4.** Prevalence of the 10 most common Clinical Classifications Software Refined (CCSR) comorbidities in the adult achondroplasia cohort compared with the prevalence extracted from the 20 most frequent CCSR comorbidities in adult non-achondroplasia controls. Comorbidities in bold font indicate overlap with the top 10 among controls.

**
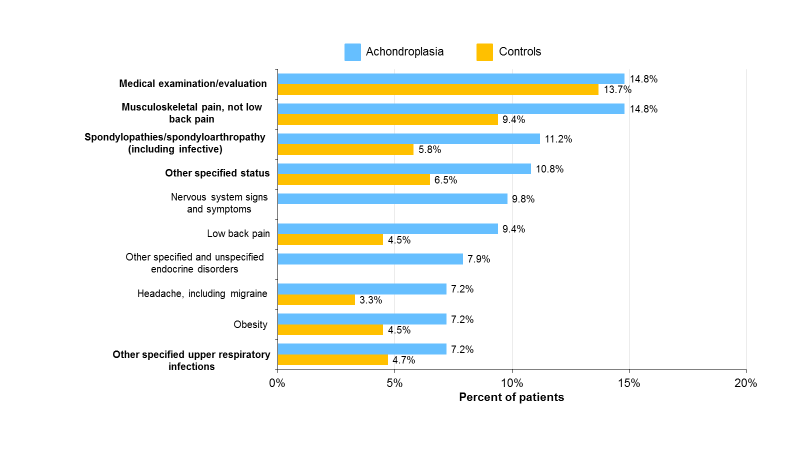
**

**Fig S5.** Healthcare resource utilization in the 12-month follow-up period among adults with achondroplasia and matched controls stratified by age. A) Inpatient stays. B) Outpatient visits. C) Home healthcare. D) Emergency room. E) Any surgery. F) Prescriptions.

**
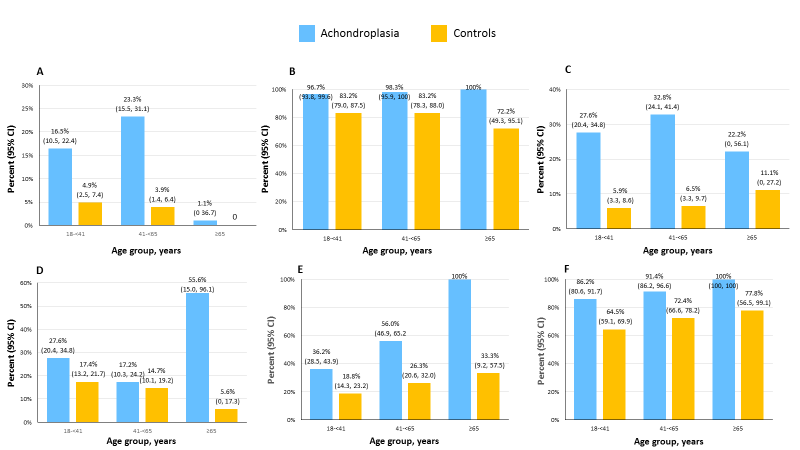
**

**Fig S6****.** Units of healthcare resource use in the 12-month follow-up period among adults with achondroplasia and matched controls stratified by age. A) Inpatient stays. B) Outpatient visits. C) Home healthcare. D) Emergency room. E) Any surgery. F) Prescriptions.

**
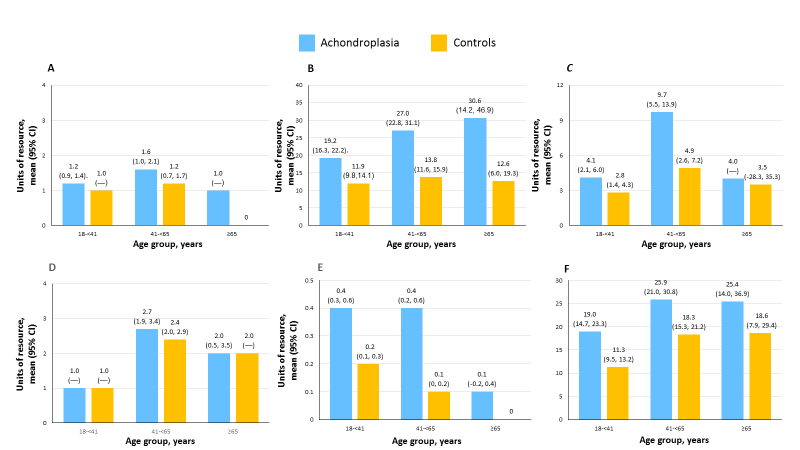
**

**Table S1.** Population attrition

| **Cohort selection step** | **Achondroplasia cohort** | **Control cohort** |
| --- | --- | --- |
| All individuals enrolled between January 2008 and December 2020 | 205,760,936 | 205,760,936 |
| With/without index diagnosis of achondroplasia with ICD-10 Q774 or with an index diagnosis of ICD-9 7564 *and* at least 1 ICD-10 Q77.4 | 2,435 | 205,745,562 |
| Exact match on year of birth and sex | — | 180,178,607 |
| With 6 months pre-index continuous enrollment | 870 | 129,270,564 |
| With 12 months post-index continuous enrollment | 609 | 39,142,948 |
| With no cancer diagnosis 6 months pre-index through 12 months post-index | 530 | 35,481,677 |
| Final cohorts after 1:2 matching | 530 | 1,060 |

**Table S2.** All-cause healthcare resource utilization costs in the 12-month follow-up period among pediatric individuals with achondroplasia and matched controls stratified by age groups.

| **Resource** | **Costs, mean±SD (95% CI), $** | | | | | | | |
| --- | --- | --- | --- | --- | --- | --- | --- | --- |
|  | **0-<2 years** | | **2-<6 years** | | **6-<11 years** | | **11-<18 years** | |
|  | **Achondroplasia**  **(n = 24)** | **Control**  **(n = 48)** | **Achondroplasia**  **(n = 53)** | **Control**  **(n = 106)** | **Achondroplasia**  **(n = 77)** | **Control**  **(n = 154)** | **Achondroplasia**  **(n = 99)** | **Control**  **(n = 198)** |
| Inpatient | 169,165±828,699  (-180,764, 519,094) | 0 | 7,813±40,138  (-3,250, 18,877) | 277±2,361  (-177, 732) | 199±1,750  (-198, 597) | 19±240  (-19, 58) | 6,622±37,073  (-772, 14,016) | 23±322  (-22, 68) |
| ER | 330±897  (-49, 709) | 227±507  (80, 375) | 482±1,245  (139, 825) | 276±773  (127, 425) | 115±329  (40, 189) | 88±376  (289, 148) | 205±813  (42, 367) | 244±1,331  (57, 430) |
| Non-ER outpatient visit | 9,056±10,680 (4,546, 13,565) | 2,073±2,092 (1,465, 2,680) | 10,481±32,534  (1,5134, 19,449) | 1,235±2,730  (709, 1,760) | 3,946±6,999  (2,358, 5,535) | 1,123±5,177  (299, 1,947) | 6,672±17,822  (3,118, 10,227) | 1,546±4,581  (904, 2,188) |
| Prescription | 5423±1,188  (41, 1,044) | 120±196  (63, 177) | 1,205±5,138  (-211, 2,621) | 233±703  (97, 368) | 1,466±6,733  (-62, 2,995) | 268±769  (146, 391) | 537±1,314  (275, 799) | 547±3,575  (46, 1,048) |
| Total | 179,093±831,390  (-171,972, 530,159) | 2,419±2,279  (1,758, 3,081) | 19,866±72,221  (-41, 39,773) | 2,013±4,546  (1,138, 2,889) | 5,727±10,625  (3,315, 8,138) | 1,499±5,301  (655, 2,343) | 14,036±48,453  (4,372, 23,700) | 2,360±6,389  (1,465, 3,255) |

CI, confidence interval; ER, emergency room; SD, standard deviation

**Table S3.** All-cause healthcare resource utilization costs in the 12-month follow-up period among adults with achondroplasia and matched controls stratified by age groups.

| **Resource** | **Costs, mean±SD (95% CI), $** | | | | | |
| --- | --- | --- | --- | --- | --- | --- |
|  | **18-<41 years** | | **41-<65 years** | | **≥65 years** | |
|  | **Achondroplasia**  **(n = 152)** | **Control**  **(n = 304)** | **Achondroplasia**  **(n = 116)** | **Control**  **(n = 232)** | **Achondroplasia**  **(n = 9)** | **Control**  **(n = 18)** |
| Inpatient | 10,158±36,235  (4,351, 15,965) | 855±5,165  (272, 1,438) | 18,631±72,730  (5,255, 32,007) | 1,134±7,299  (190, 2,078) | 9,948±29,845  (-12,992, 32,889) | 0 |
| ER | 472±1,657  (206, 737) | 210±664  (135, 285) | 261±909  (94, 428) | 238±897  (122, 354) | 740±996  (-26, 1,506) | 33±138  (-36, 101) |
| Non-ER outpatient visit | 4,969±7,886  (3,705, 6,232) | 2,049±5,738  (1,401, 2,697) | 7,377±9,769  (5,580, 9,175) | 3,105±7,010  (2,198, 4,011) | 4,296±3,162  (1,866, 6,726) | 4,104±10,960  (-1,347, 9,554) |
| Prescription | 1,372±3,717  (776, 1,968) | 1,372±3,717  (776, 1,968) | 1,609±5,660  (68, 2,650 | 1,706±7,176  (778, 2,634) | 5,592±13,206  (-4,559, 15,743) | 2,054±6,559  (-1,207, 5,316) |
| Total | 16,871±43,632  (9,879, 23,863) | 3,962±10,906  (2,731, 5,193) | 27,850±75,311  (13,999,41,700) | 6,150±15,318  (4,168, 8,132) | 20,274±29,907  (-2,714, 43,264) | 6,191±12,941  (-245, 12,626) |

CI, confidence interval; ER, emergency room; SD, standard deviation
